# Investigating the Role of Nutrition in Enhancing Immunity During the COVID-19 Pandemic: Twitter Text-Mining Analysis

**DOI:** 10.2196/47328

**Published:** 2023-07-10

**Authors:** Kavitha Shankar, Ranganathan Chandrasekaran, Pruthvinath Jeripity Venkata, Derek Miketinas

**Affiliations:** 1 Department of Nutrition and Food Sciences Texas Woman's University Institute for Health Sciences Houston, TX United States; 2 Department of Information and Decision Sciences University of Illinois at Chicago Chicago, IL United States

**Keywords:** social media, nutrition discourse, text mining, immunity building, food groups, Twitter, nutrition, food, immunity, COVID-19, diet, immune system, assessment, tweets, dieticians, nutritionists

## Abstract

**Background:**

The COVID-19 pandemic has brought to the spotlight the critical role played by a balanced and healthy diet in bolstering the human immune system. There is burgeoning interest in nutrition-related information on social media platforms like Twitter. There is a critical need to assess and understand public opinion, attitudes, and sentiments toward nutrition-related information shared on Twitter.

**Objective:**

This study uses text mining to analyze nutrition-related messages on Twitter to identify and analyze how the general public perceives various food groups and diets for improving immunity to the SARS-CoV-2 virus.

**Methods:**

We gathered 71,178 nutrition-related tweets that were posted between January 01, 2020, and September 30, 2020. The Correlated Explanation text mining algorithm was used to identify frequently discussed topics that users mentioned as contributing to immunity building against SARS-CoV-2. We assessed the relative importance of these topics and performed a sentiment analysis. We also qualitatively examined the tweets to gain a closer understanding of nutrition-related topics and food groups.

**Results:**

Text-mining yielded 10 topics that users discussed frequently on Twitter, viz proteins, whole grains, fruits, vegetables, dairy-related, spices and herbs, fluids, supplements, avoidable foods, and specialty diets. Supplements were the most frequently discussed topic (23,913/71,178, 33.6%) with a higher proportion (20,935/23,913, 87.75%) exhibiting a positive sentiment with a score of 0.41. Consuming fluids (17,685/71,178, 24.85%) and fruits (14,807/71,178, 20.80%) were the second and third most frequent topics with favorable, positive sentiments. Spices and herbs (8719/71,178, 12.25%) and avoidable foods (8619/71,178, 12.11%) were also frequently discussed. Negative sentiments were observed for a higher proportion of avoidable foods (7627/8619, 84.31%) with a sentiment score of –0.39.

**Conclusions:**

This study identified 10 important food groups and associated sentiments that users discussed as a means to improve immunity. Our findings can help dieticians and nutritionists to frame appropriate interventions and diet programs.

## Introduction

### Background

The global COVID-19 pandemic has put a spotlight on the critical role that nutrition has in supporting the human immune system, and in building resilience and resistance against infections with viral and bacterial pathogens. Per the Academy of Nutrition and Dietetics Foundation, food as medicine is “a reaffirmation that food and nutrition play a role in sustaining health, preventing disease, and as a therapy for those with conditions or in situations responsive to changes in their diet” [1]. Diet plays a critical role in overall health and wellness, in addition to serving as a tool to combat diseases. The nutritional guidelines by the World Health Organization suggest a well-balanced diet enriched in vitamins, minerals, dietary fiber, proteins, and antioxidants to build immunity against the novel coronavirus [2]. Macronutrients like proteins are essential for producing antibodies, and micronutrients like vitamins and minerals with anti-inflammatory and antioxidant properties can help build a line of defense against the virus [3]. Therefore, a balanced and healthy diet rich in such nutrients may be critical in reducing the risk for infectious diseases caused by viruses such as the SARS-CoV-2 virus [4].

In the wake of COVID-19 and the ensuing government-mandated lockdowns, many individuals turned to social media for information and guidance about the pandemic. Though government agencies such as the Centers for Disease Control and health care agencies provided detailed information on the pandemic [5] via press releases and mainstream media, most of the public actively used social media platforms [6] for information on the disease and ways to deal with the crisis. From doling out often unvalidated medical advice to distributing accurate information [7], social media users took the center-stage as information disseminators [6]. Social media platforms such as Twitter and Facebook became fertile grounds for individuals to express their opinions, give and get information, share their experiences, and engage in knowledge exchanges about the novel coronavirus. These platforms became rich repositories of information about the pandemic. Using this fairly large data sets on social media, studies have applied machine learning and text mining techniques to assess public perceptions, attitudes, sentiments, and emotions pertaining to COVID-19 pandemic [8-10], self-reported experiences [11-13], clinical trials of vaccines, vaccination mandates [14-16], and side effects [17]. Another group of studies has also examined the spread of misinformation about the pandemic through social media [18,19]. These studies have collectively highlighted the rich source of information that is available in social media that could be harnessed using advanced machine learning approaches that can facilitate the design of appropriate health interventions and promotion campaigns.

Though traditional web-based platforms such as websites and health portals have been a popular medium for information dissemination on food, diet, and nutrition [20], the popularity and reach of social media sites have made them attractive platforms to actively exchange and disseminate nutrition and diet-related information. Previously, a few sources like experts, reporters, and writers served as content producers, while media outlets primarily decided on sharing that content. However, social networks have drastically altered the way in which information is produced, delivered, and consumed. Individual users of social media platforms have become content creators, producers, and disseminators. In addition to experts, the common public has also been actively engaged in discussing nutrition-related information on social media platforms [21]. Social media users have also been documenting their choices about food and diet [22]. A growing number of studies have examined the nutrition-related information that is being discussed on social networks and the dynamics underlying them [23-25]. For example, previous studies have examined social media data to assess public sentiments about various kinds of foods and their nutritional content [26], healthy diet and lifestyle [27], artificial sweeteners [28], and gluten-free food [29]. Text-mining and analytics are becoming quite prevalent in health care related studies [30]. Joining this cohort, this study seeks to examine public perceptions and information exchanges on social media about the role of nutrition and diet to fight the pandemic. Using text-mining techniques, we investigate tweets about nutrition and diet to identify the nature of the information that was disseminated and exchanged during the pandemic. Our specific goal is to identify food groups or types that users perceived were beneficial or harmful to improving their immunity during the pandemic.

Our study makes 3 distinct contributions. First, we identify and prioritize the food groups and diet types that Twitter users believe can help build a stronger immune system against SARS-CoV-2. Second, unlike previous studies that relied on manual annotation to content analyze social media posts about nutrition [21], we used advanced natural language processing techniques. In response to the recent calls to advance nutrition research using artificial intelligence tools [31,32], we used sophisticated text mining algorithms on a relatively large data set of tweets to investigate our research questions. Third, our study provides evidence of increased nutrition literacy and knowledge among Twitter users. Previously, we had a limited understanding of the global public’s awareness about nutrition. By extracting Twitter users’ opinions and views, we gather collective wisdom on food types that can be beneficial in fighting the novel coronavirus.

### Objective

Our research goal was to examine Twitter posts to understand what the public feels about foods and diet in improving immunity against the novel coronavirus. We analyzed the extent of Twitter discourse on different food groups, assess their relative importance, and attitudes of users toward these food groups.

## Methods

### Data Collection

For collecting past tweets based on keywords, we considered options including Rtweets, Tweepy, Twitter application programming interface (API), and TWINT. However, we chose TWINT due to its ability to bypass the limitations of Twitter’s API regarding time frame limitations for collecting tweets. TWINT allows for historical tweet collection through its advanced search functionality and can collect tweets quickly and in bulk [33]. Additionally, TWINT provides more control over the search parameters, including filtering by date range and language. Sample keywords used for the data gathering were: nutrition, eat, drink, food, diet, immune, or immunity. The search excluded Tweets that were ategoryized as retweets. Tweets published between January 1, 2020, and September 1, 2020, were gathered and downloaded into a text file in comma separated (csv) format. As our primary interest was to examine the public’s posts about food, the source of tweets examined that it was an individual and not a commercial entity making the tweet. Our final data set comprised 71,178 tweets.

### Ethical Considerations

As we used publicly available tweets in this study, no human subjects review was required. However, we recognize the importance of ethical considerations in social media research, and we took steps to ensure the privacy and confidentiality of the Twitter users whose tweets were analyzed. We have presented aggregated and unidentified data to avoid the identification of individual users

### Data Preprocessing

In order to prepare the tweets for analysis, we used several data cleansing and preprocessing techniques. First, we used the Stanford Natural Language Toolkit and its lemmatizer algorithm to lemmatize the tweets. This algorithm helps to derive the base or root form of a word from its inflectional forms. For example, “foods” and “food” would both be lemmatized to “food.” This process ensured that different variations of the same word were consolidated, reducing noise in the data set and allowing us to focus on the underlying themes and topics.

**Figure 1:**
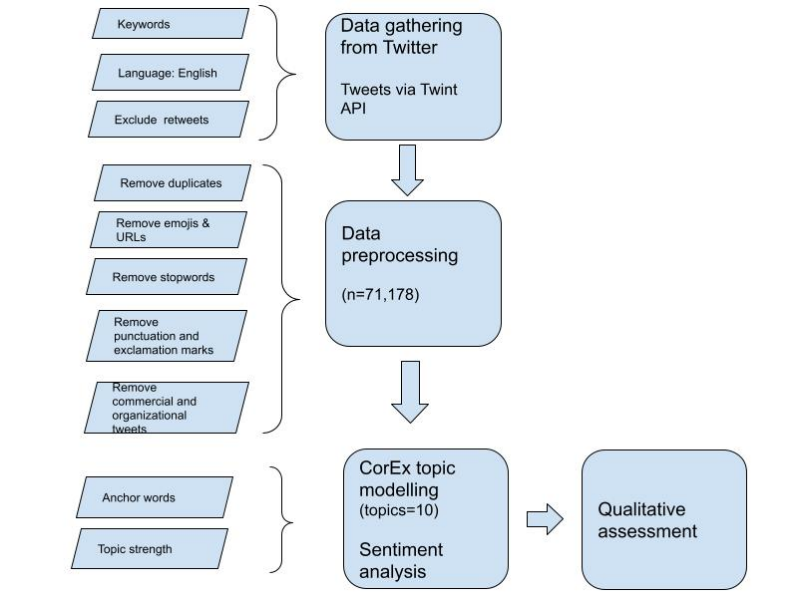
Study workflow.

Next, we used the Natural Language Toolkit stop words dictionary to remove stop words. Stop words are common words that do not carry much meaning, such as “the,” “and,” “is,” and so on. By removing these words, we were able to focus on the more meaningful words in the tweets. Finally, we conducted additional cleaning of the tweets to remove unwanted characters and punctuation marks. This included removing repetitive words, @ symbols, and other special characters that were not essential to our analysis. By applying these preprocessing steps, we were able to clean and prepare the data for subsequent analysis, allowing us to extract insights and patterns that were relevant to our research questions. The workflow for the data collection, preparation, and analysis is depicted in Figure 1.

### Modeling Technique and Topic Discovery

To explore the topics or themes that underlie nutrition-related tweets, we used topic modeling, a popular text-mining method for identifying latent patterns of words in a large corpus of unstructured textual documents [34,35]. Topic modeling seeks to extract a smaller number of topics that are commonly embedded across a large collection of textual data. A topic model algorithm scans a set of documents, examines co-occurrences of words and features, and automatically identifies clusters of words that best characterize those documents. Topic modeling helps in describing a large text data set using a moderate number of themes that permeate throughout the texts. The extracted topics help in attaining a rich characterization of the entire corpus of textual data.

Among the many approaches to topic modeling, Latent Dirichlet Allocation (LDA) is a popular algorithm that has gained wide traction [36]. LDA is an unsupervised algorithm where no prior knowledge of topics is needed. By tuning the LDA parameters, one can explore different topic formations and document clusters. LDA is based on detailed assumptions and careful specification of hyperparameters. LDA outcomes can be hard to interpret and can drastically vary based on choice of parameters. In LDA, some topics could get washed out by other topics with greater presence in the corpus [37].

Recent advances in topic modeling have produced semisupervised approaches that allow the injection of some prior domain knowledge using “seed words” into the topic model. This allows flexibility to steer the algorithm toward some topics that are of interest, while also allowing room to uncover unknown topics. A semisupervised topic modeling algorithm—CorEx (Correlation Explanation), learns maximally informative topics through an information-theoretic framework [38]. CorEx allows the incorporation of domain knowledge through user-specified anchor words that guide the model toward specific topics. Given our interest in using US Department of Agriculture (USDA) defined food groups and Dietary Guidelines for Americans, CorEx was the technique that was most appropriate for our research. CorEx has been used in prior studies that have examined social media data on dietary supplements [39], eating disorders [40], and COVID-19 vaccinations [15].

We used the USDA food groups [41] and associated keywords as a starting point and iteratively refined the number of topics that were generated by the CorEx algorithm. This led to the identification of newer topics such as specialty diets including vegetarian and vegan diets and avoidable foods. CorEx ultimately yielded 10 topics underlying the corpus of tweets. Using the topic strength score provided by CorEx, each tweet in our data set was categorized as belonging to the topic that best reflected the tweet content. We computed the frequency of occurrences of the 10 topics in our tweets and tabulated them to assess the relative importance of the topics.

Sentiment Analysis involves using computational methods to determine whether the language used in a given text has a positive, negative, or neutral tone and sentiment. In this study, we used VADER (Valence Aware Dictionary and Sentiment Reasoner), a popular Python-based sentiment analysis tool, to assess the polarity of sentiments expressed in the reviews. VADER generates a compound score by summing up the positive, negative, and neutral polarity scores, which are then normalized between –1 (most extreme negative sentiment) and +1 (most extreme positive sentiment). VADER has been widely used in prior studies examining health-related tweets and we used it to categorize our tweets as carrying positive, negative, or neutral sentiments. Finally, to get an in-depth understanding of each of the topics, we did a qualitative content analysis of tweets underlying each of the 10 topics. Both CorEx topic modeling and VADER sentiment analysis were performed using Python software.

## Results

### Overview

**Figure 2:**
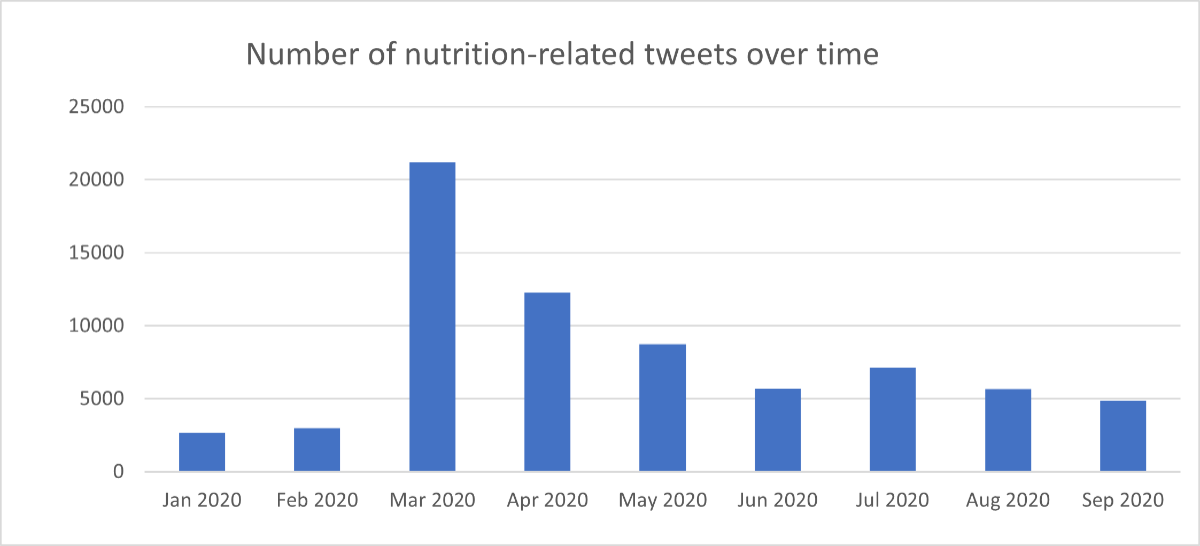
Trends in number of nutrition-related tweets (January-September, 2020).

The temporal distribution of nutrition-related tweets during the time period of examination is shown in Figure 2. It is not surprising that nutrition-related tweets peaked in March 2020 when the WHO declared COVID-19 as a global pandemic. We found about 5000 tweets or more in every subsequent month until September 2020. These trends indicate a burgeoning interest in nutrition-related discourse as a means to build immunity during the initial phases of the pandemic.

The results of the CorEx topic modeling in terms of 10 topics and important keywords that were associated with the topics are presented in Table 1. Based on the keywords, we identified the following topics: Protein, Whole Grains, Fruits, Vegetables, Dairy-related, Spices and Herbs, Fluids, Supplements, Avoidable Foods, and Specialty Diets. In addition to the initial topics that were from USDA food group gallery [42], our topic modeling yielded 5 additional topics that were discovered from the mining of tweets.

**Table 1:** Topics and keywords.

Topics	Representative keywords
Proteins	Egg, protein, meat, chicken, fish, beef, lamb, pork, ham
Whole grains	Rice, bread, wheat, corn, millet, oats, oatmeal, quinoa, flour
Fruits	Lemon, lime, citrus, juice, orange, berry, banana, apple, nuts, kiwi
Vegetables	Veggie, greens, leafy, cabbage, spinach, onion
Dairy related	Milk, yogurt, cheese, dairy, butter, kefir
Spices and herbs	Herb, pepper, cinnamon, mint, turmeric, garlic, ginger, seeds
Fluids	Water, hydrate, tea, smoothie, honey, fluid, warm, hot
Supplements	Vitamins, zinc, supplements, iron, omega, antioxidants, mineral, selenium
Avoidable foods	Beer, soda, wine, smoke, alcohol, fast food, junk, tobacco
Specialty diets	Vegan, vegetarian, herbal, ayurvedic, alkaline, keto

### Frequency of Topics and Their Relative Importance

To identify the topics associated with a tweet, we used CorEx’s document topic matrix, which lists the probabilities of a topic being present in a tweet. Next, we evaluated the significance of each topic by analyzing the count of occurrences of those topics in the repository of tweets. It should be noted that a tweet could contain references to more than one topic. Table 2 displays the frequency of occurrences of nutrition-related topics in our data set.

Based on our analysis, supplements are the most commonly referenced topic among Twitter users, with over 33.6% (23,913/71,178) of tweets mentioning them as a way to improve immunity. Intake of fluids was mentioned in nearly 24.85% (17,685/71,178) of the tweets, while 20.80% (14,807/71,178) of the tweets referenced fruits as a way to boost immunity against the novel coronavirus. Spices and herbs were mentioned in 12.25% (8719/71,178) of the tweets, and vegetables were referenced in 10.47% of the tweets. Interestingly, 12.11% (8619/71,178) of the tweets suggested avoiding certain foods. Specialty diets were mentioned in 9.79% (6968/71,178) of the tweets, and intake of proteins was suggested in 8.92% (6352/71,178) of the tweets. Dairy-related items and whole grains were relatively less frequently mentioned in the tweets, with only 6.42% (4567/71,178) and 5.9% (4203/71,178)of tweets referencing them, respectively.

Results of the sentiment analysis of tweets are provided in Table 3. For 7 topics, over 80% (57,013/71,178) of tweets that referenced these topics exhibited positive sentiment, reflecting the favorable opinion of Twitter users toward the food-related items associated with these topics; 87.55% (20,935/23,913) of the tweets associated with supplements exhibited a positive sentiment. It was not surprising that 84.31% (7267/8619) of tweets that referenced “avoidable foods” carried a negative sentiment. Close to 20% (1270/6352) of tweets about proteins and 18.34% (771/4203) of tweets about whole grains exhibited negative sentiments. Supplements had the highest average compounded sentiment score (0.58), followed by fruits (0.537) and spices and herbs (0.518). Avoidable foods had a negative, average compounded score of –0.393.

**Table 2:** Nutrition-related topics and frequency of occurrences.

Topics	Tweets, n (%)
Proteins	6352 (8.92)
Whole grains	4203 (5.90)
Fruits	14,807 (20.80)
Vegetables	7454 (10.47)
Dairy related	4567 (6.42)
Spices and herbs	8719 (12.25)
Fluids	17,685 (24.85)
Supplements	23,913 (33.60)
Avoidable foods	8619 (12.11)
Specialty diets	6968 (9.79)

**Table 3:** Results of sentiment analysis using VADER (Valence Aware Dictionary and Sentiment Reasoner).

Topics	Tweets with positive sentiment, n (%)^a^	Tweets with negative sentiment, n (%)	Tweets with neutral sentiment, n (%)	Average compounded sentiment score^b^
Proteins	4874 (76.73)	1270 (19.99)	208 (3.27)	0.411
Whole grains	3193 (75.97)	771 (18.34)	239 (5.69)	0.407
Fruits	12,596 (85.07)	1863 (12.58)	348 (2.35)	0.537
Vegetables	6184 (82.96)	1088 (14.60)	182 (2.44)	0.501
Dairy-related	3748 (82.07)	621 (13.60)	198 (4.34)	0.481
Spices and herbs	7338 (84.16)	984 (11.29)	397 (4.55)	0.518
Fluids	14,208 (80.34)	3083 (17.43)	394 (2.23)	0.455
Supplements	20,935 (87.55)	2584 (10.81)	394 (1.65)	0.584
Avoidable foods	1097 (12.73)	7267 (84.31)	255 (2.96)	–0.393
Specialty diets	5575 (80.01)	887 (12.73)	506 (7.26)	0.469

^a^Represents the number and percentage of tweets with a positive sentiment score from Valence Aware Dictionary and Sentiment Reasoner analysis.

^b^Average of sentiment score where –1 represents most extreme negative sentiment and +1 represents most extreme positive sentiment.

## Discussion

### Principal Findings

Our study used text mining via CorEx topic modeling to analyze nutrition-related tweets and gain insights into how Twitter users discussed building immunity against the novel coronavirus. By identifying topics and food groups and assessing their relative significance, we were able to better understand the information exchanged among Twitter users on this topic. This research project further demonstrates the effectiveness of text mining and topic modeling as a tool for extracting valuable insights from large amounts of social media data.

Applying CorEx topic modeling methods, we were able to identify 10 topics or food groups. The most frequently discussed topic was about supplements. Other studies examining internet searches using Google Trends have also noted increased public interest in dietary supplements during the pandemic [43]. Our findings about the importance of supplements are further validated by the sudden spike in sales of supplements during the initial months of the pandemic. Before the pandemic, dietary supplement sales in the United States grew by 5% (US $345 million) in 2019 compared to the previous year. However, during the first wave of the pandemic, there was a significant surge in sales, with a 44% increase (US $435 million) in sales during the 6 weeks leading up to April 5, 2020, compared to the same period in 2019 [44]. This surge in sales of supplements has been driven by heightened interest among the general public to use them as a mechanism to build immunity against the virus. Our study corroborates this increased interest in supplements during the initial months of the pandemic. When we examined the tweets closely, we found specific suggestions and recommendations from users about the kind of supplements for immunity building. For instance, one tweet mentioned, “Prevention is better than cure. Take vitamins to strengthen your immune system, suggested vitamin C, B, E, and zinc.” Another user tweeted, “For Coronavirus, I would suggest that you boost your immune system first and lay off unhealthy processed food and sugar. Start supplementing with Zinc, at least 10,000 IU’s of D3 with some K2 With vitamin C.” Another user said, “To maintain my healthy immune system, I take 1000mg of Vitamin C and 50mg of Zinc.” Such a public frenzy about supplements was accompanied by the emergence of supplements that were advertised as products with the potential to prevent or cure COVID-19, leading to calls for more regulatory oversight to curb misleading or falsified information about supplements [44]. Warnings about such newer supplements were seen in the tweets we examined. One user cautioned, “People can be so gullible and use the money they’ll use to feed to buy supplements that may not be beneficial to them.” Another user mentioned, “As COVID19 spreads, having an optimally functioning immune system is important, but do not rush to buy supplements and vitamins that promise to enhance your immune system.”

Our analysis indicated fluids to be the next important topic that Twitter users discussed. The human body needs to stay hydrated for proper functioning as it is made up of about 60% water. Water and hydration are essential for maintaining a strong immune system. The human body needs water to carry nutrients and oxygen to cells, remove waste products, and regulate body temperature. During the first few months of pandemic, the importance of hydration and water as a means to strengthen the immune system was discussed by several users on Twitter. “Drink a lot of water to hydrate your cells. Dehydrated cells are easily collapsed and prone to viral takeover,” said a user. Another one remarked, “Everyone, pls take care of urself !! stay safe and hydrated all the time. Drink lots of water.” In addition to water, many users mentioned the benefits of herbal and other teas as exemplified by this tweet: “Regular intake of Ashitaba tea (high in antioxidants) can help strengthen your immune system.” Users had mentioned many specialty teas such as “echinacea tea,” “Chinese red date pear tea,” “ginger tea,” “turmeric tea,” “chamomile tea,” and “ginseng tea” as effective in building immunity. Green tea was also mentioned by many users. Users also mentioned consuming a multitude of teas. “I drink 4 teas this morning. A hibiscus tea for blood pressure, a probiotic ginger tea for my gut health, a lemon vitamin C tea for immune support, and a green tea for overall health. The lemon and ginger have the best taste.” Honey was also cited as an important intake to build immunity. A user tweeted, “Honey is a prebiotic, good for Immunity and overall health. It is a great source of energy, is rich in antioxidants, and full of vitamins and minerals.”

According to our analysis, fruits emerged as the next, most frequently mentioned sources of immunity by Twitter users. Among the fruits, citrus was often cited for its immunity-building properties due to it being a source of Vitamin-C. As a user tweeted, “Eat citrus fruits for essential, immune boosting Vitamin C, an antioxidant that helps heal wounds; keep gums healthy. RED oranges have 34 x more vitamin A than regular oranges.” Another user remarked, “adding citrus fruits to your diet regimen helps to provide your body with antioxidants or vitamin C (which boosts your immune system). here is a list of citrus fruits: grapefruit, oranges.” Several tweets mentioned the benefits of lemons. One user mentioned: “Eat natural vitamin C to boost your immune system; cut a lemon, pour warm water on it, and drink throughout the day.” Another tweet said, “Lemons contain a high amount of vitamin C which is important for many functions, including immune and heart health. They also have folate, calcium, magnesium, and copper.” In addition to these fruits, apple, papaya, kiwi, pineapple, and berries were some additional ones that found frequent mention in the tweets. The topic of vegetables was also of considerable interest to Twitter users, though the mentions of vegetables were comparatively less than that of fruits. Among the vegetables, green leafy ones like spinach, kale, and cabbage, and carrots, broccoli, and cauliflower were suggested for building immunity. As one tweeter remarked, “Add foods like sweet potatoes, tomatoes, red peppers, and citrus fruits to your meals as they contain vitamin C, which plays an important role in the immune system and energy levels.”

Spices and herbs were another frequently discussed topic that emerged from our CorEx analysis. Recent studies have also highlighted the role of spices and herbs in improving the immune system to combat the novel coronavirus [45-47]. In our analysis, we found Twitter users to mention spices and herbs from medicinal plants that have been used in traditional medical practices in India and China, as potential therapeutic agents. Consuming ginger and garlic was suggested by many users. A tweet mentioned, “I drink ginger, mint, and honey everyday. Steep it in a glass jar. Boosts your immune system, kills anything that should not be there.” Another user said, “Eat foods that help your immune system. Ginger, garlic, turmeric, cayenne.” Other common herbs and spices that were mentioned include cinnamon, ginseng, ashwagandha, cumin, black pepper, turmeric, coriander seeds, tulsi, thyme, and so on. One user mentioned, “Beef up your immune system with extra zinc and curcumin, and when you interact with people, drink a hot saffron tea or tulsi to put viruses in your gut where they die.” Interestingly, some users had also shared short cooking recipes suggesting how spices and herbs can be incorporated into the regular diet. As one user remarked, “Try making this Ayurvedic Decoction with ginger, black pepper, cinnamon, coriander seeds, and black raisins. This drink is packed with phytonutrients and antioxidants.”

Twitter users not only discussed what foods to eat but also highlighted several foods that should be avoided. Numerous tweets focused on the harmful effects of specific foods, drugs, and their production processes. Users suggested avoiding processed and junk foods and sugared products. Many cautioned against smoking and consuming alcohol as they could weaken the immune system. As one user remarked, “I suspect a chunk of them have weak immune systems: sugared coffee from Starbucks, alcohol, smoking, junk food, staying indoors.” Another user tweeted, “Just a reminder for all those taking Vitamin C to boost their immune systems. Drinking alcohol, smoking, and sugary drinks all deplete your Vitamin C levels.” Another tweet mentioned, “Do NOT drink alcohol daily. It weakens OUR biological immune system…alcohol depletes Vitamin B1, B12, D3, K, weakens liver, metabolism, etc.” Though many users warned against drinking alcohol, some users suggested consuming red wine as a means to boost immunity. Sugared beverages and soda were also mentioned as avoidable foods in many of the tweets. Some users advised against eating raw, uncooked food, as exemplified by this tweet. “She also advised to refrain from eating raw (raw fish, raw chicken, and eggs) and junk food in times of coronavirus.”

From our analysis, we also found specialty diets to be a frequently mentioned topic on Twitter as a means to build immunity to the coronavirus. Many Twitter users mentioned the benefits of a vegetarian and vegan diet for improving immunity. One tweet remarked, “Vegetarian diet is associated with higher fiber, vitamins, folic acid, magnesium, and phytochemicals which helps in boosting Immunity.” Another user said, “Vegetarians typically have a higher intake of fruit and vegetables, antioxidant nutrients, and phytochemicals, for adequate immune function.” Naturopathy and keto diets were also discussed by the users in our data set. One user mentioned, “Go for the lemon water instead of the coke and a straight keto diet high in nutrient and immune support vitamins. That’s a tough concept for some to be able to appreciate.” Extolling naturopathy, one user shared, “We prefer home cooked food—vegetarian food. We drink normal tap water, not mineral water. We use natural products for mild health issues which also enhance our immunity. We mostly live in an unprotected environment without air conditioners or heaters. Lifestyle is a major factor why our immunity is more.” We also found tweets that referred to plant-based diets, ayurveda diets, and fat-free diets that were suggested by some users. Here is an illustrator tweet about the benefits of a plant-based diet. “Plant-based diets offer a functional take on a healthier lifestyle. Adding more plants to your diet ensures that your body receives plenty of antioxidants which help in your immune system and reduce your risk of disease.”

The other topics that our CorEx analysis revealed were about proteins, dairy-related products, and whole grains. Many users mentioned consuming eggs, chicken, and fish to improve protein intake, as can be seen from the following tweet: “Chicken and eggs are a great source of protein. Vitamin D in chicken helps remove free radicals and fights off respiratory tract infections. You can boost your immune system by adding chicken in your daily diet and have a meal full of nutrients during the pandemic.” However, another set of users also suggested lean protein and lesser consumption of protein. As an example, a user mentioned, “Ideally we want to get our nutrition through the food we eat-healthy whole grains, vegetables, lean protein, and healthy fats. MOST Americans do not eat well enough to get the nutrients they need.” Another one tweeted, “What do I mean by Healthy Diet? Most of us tend to think that taking lots of carbohydrates and proteins and at times less proteins or even none is healthy. But this is wrong, our bodies need more of vitamins for immunity.” Among the tweets that mentioned whole grains, the often-mentioned were oats, millets, lentils, and wheat. One user mentioned, “It will be of great importance and act as a perfect immune booster as a 100 gram raw millet provides about 1580 kilojoules of food energy and is a rich source of protein, several B vitamins, dietary fibers, and minerals.” Among the diary-related items, cheese was mentioned by users as a rich source of Vitamin D, A, and K. One tweet said, “Vitamin A keeps eyes, skin and immune system healthy. Where do you find it? Liver, some fish, milk, and cheese.”

It is important to note that our results convey the overall perceptions and viewpoints of Twitter users. Our findings offer valuable insights into the experiences and thoughts of users regarding particular food groups and items that they deemed helpful or detrimental to bolstering immunity against the COVID-19 pandemic. Information shared on Twitter may contain inaccuracies and may not align with the views of the medical or dietician community. Nonetheless, the collective opinions and attitudes of the general public provide valuable information on dietary practices during the pandemic, when people were searching for ways to cope with the crisis.

User-generated content on Twitter can be an effective way to disseminate and exchange nutrition-related information, provide recommendations and improve awareness among the public. Our findings indicate that users have a favorable opinion of supplements, fruits and vegetables, fluids, and certain herbs and spices. Conversely, our results reveal negative user evaluations of sugar, alcohol, junk, and processed foods. Our study has significant implications for food companies, policy makers, clinicians, and the dietician community regarding the types of foods that are preferred and disliked by the public. Additionally, our results highlight the increased awareness of nutrition during the COVID-19 pandemic, which should be beneficial for nutritionists and dieticians in designing appropriate intervention campaigns to promote healthy eating and lifestyle.

### Limitations and Future Work

It is important to acknowledge that our findings have some limitations. Our analyses did not include the perspectives of users who do not use Twitter, which may limit the generalizability of our results. Furthermore, we did not examine any user-specific information to identify differences in tweets based on demographic characteristics. We also did not examine if the nature of nutrition-related tweets differed based on the geographical locations of users. Future studies could focus on tweets originating from specific geographical locations or countries, or those exclusively made by dieticians and nutritionists, and compare them to those of the general population to explore similarities and differences. This could provide a more comprehensive understanding of nutrition-related discussions on Twitter. Another limitation of this study pertains to the choice of specific keywords used for gathering the tweets. The list of selected keywords may have been incomplete. Our source of information was only Twitter and future studies could consider other social media platforms like Instagram and Facebook, or YouTube.

We used CorEx to perform semisupervised topic modeling. Our choice of CorEx was guided by its ability to discover overlapping and hierarchical topics, where domain knowledge and human inputs can be incorporated to improve its performance. Future work could consider using BERTopic which uses the pretrained Bidirectional Encoder Representations from Transformers (BERT) model as a feature extractor and apply clustering algorithms to identify latent topics in a text repository. BERT-topic also allows for the incorporation of human inputs such as topic labels or domain knowledge [48]. Our study did not focus on any differences in nutrition-related tweets based on geographies. Future work could examine regional differences in nutrition-related tweets.

### Conclusions

This study provides insights into the information-sharing behaviors of Twitter users on nutrition-related content during the initial months of the COVID-19 pandemic. Using text mining of a fairly large corpus of tweets, we identified 10 important topics that users discussed as a means to bolster immunity against the novel coronavirus. Our study also identified favorable and unfavorable attitudes of users toward specific food groups. Our study provides practical and timely insights for dieticians, nutritionists, and other health care professionals to frame appropriate programs and interventions, especially during times of the pandemic.

## Abbreviations

API: application programming interface
BERT: Bidirectional Encoder Representations from Transformers
CorEx: correlation explanation
LDA: Latent Dirichlet Allocation
USDA: US Department of Agriculture
VADER: Valence Aware Dictionary and Sentiment Reasoner

## Data Availability

Due to Twitter’s data policies, only the tweet-ids of the tweets used in this study will be made available on request. Interested researchers can access use any hydration software for Twitter to obtain the full tweets and related information.
